# Editorial: Wearable robotics in the rehabilitation continuum of care: assessment, treatment and home assistance

**DOI:** 10.3389/fnbot.2023.1305786

**Published:** 2023-10-20

**Authors:** Emilio Trigili, Sandra Hirche

**Affiliations:** ^1^The BioRobotics Institute, Scuola Superiore Sant'Anna, Pisa, Italy; ^2^Department of Excellence in Robotics and AI, Scuola Superiore Sant'Anna, Pisa, Italy; ^3^Chair of Information-Oriented Control, Department of Electrical and Computer Engineering, Technical University of Munich, Munich, Germany

**Keywords:** wearable robotics, rehabilitation robotics, robotic assessment, functional electric stimulation (FES), exoskeletons

Global population aging is posing long-term challenges to societal welfare and sustainability. The prevalence of age-associated chronic diseases is causing a growing demand for physical and cognitive rehabilitation. Healthcare providers are faced with the challenge of guaranteeing a continuum of care after hospital discharge, and the management and delivery of outpatient and home rehabilitation has become critical (Gonzaga et al., 2023).

Wearable robotics can support people affected by neurological conditions in recovering their motor functions by aiding therapists in providing customized, task-specific rehabilitation training, or by augmenting human movement capabilities in activities of daily living (ADLs). The number of commercially available wearable robotics is rising across different application domains, predominantly in the healthcare and industry sectors. Nevertheless, several open challenges remain regarding actuation, sensing, and control, which limit the wide adoption of these devices outside the controlled laboratory environment (Babič et al., 2021).

Exoskeletons made of physically compliant structures, usually referred to as *soft* exoskeletons or *exosuits*, are promising for their capability to assist users with mild to moderate impairments while maintaining a lightweight and compact structure. Maldonado-Mejía et al. presented a fabric-based hand exoskeleton with pneumatic actuation (ExHand), designed for assisting grasping tasks in ADLs. The capability of the device to maintain stable contact with different objects was verified on 10 participants without any hand impairments. Di Natali et al. developed a modular lower-limb exosuit for walking assistance (XoSoft). The exosuit uses soft pneumatic *quasi-passive* actuators, which can modulate the forces generated by the deformation of an elastic tendon via a variable stiffness textile-based clutch. This approach mimics the behavior of the human muscle and tendons and allows the injection of positive energy into the gait cycle without requiring powerful actuators.

Over the last few years, hybrid neuroprostheses combining low-power robotic actuation with functional electrical stimulation (FES) have also been proposed. Such hybrid systems provide biomimetic assistance by distributing the power resources between the user's muscles and the robotic actuator. A recent review paper analyzed the efficacy of hybrid neuroprostheses employed in randomized controlled trials for upper-limb impairment after stroke, showing their positive effects in the recovery of upper-limb motor function (Höhler et al., 2023). Still, most of the current hybrid devices use both actuation types to address separate functions for each (e.g., distal or proximal joint actuation). Dunkelberger et al. presented a controller based on model predictive control for a hybrid upper-limb powered exoskeleton to assist individuals with spinal cord injuries. The controller was designed to provide an optimal distribution of power at the same joint, favoring FES over robotic actuation to assist in tracking movements.

Physical training, either via FES or via robotic devices, can also be combined with cognitive training by means of serious games based on virtual or augmented reality. Such hybrid combinations are suggested to increase patients' engagement, motivation, and adherence to the treatment. Höhler et al. recruited 18 patients after stroke in a randomized crossover trial to investigate the feasibility and benefits of combining serious games with contralaterally electromyography-triggered FES. The results of this study also provide valuable insight into the potential translation of such systems to the home environment.

To ensure a continuum of care from hospital to home rehabilitation, it is paramount to develop devices that are intuitive, portable, and easy to use. These devices should be designed in a way that allows them to be worn and used without the supervision of the therapist, gathering at the same time quantitative measures to monitor the progress of the therapy. Bressi et al. investigated the use of a robotic end-effector type device (iCONE, Heaxel, Italy) for the home-based rehabilitation of chronic stroke patients. The study encourages the exploration of possible correlations between the clinical evaluation scales and the metrics obtained via the robot sensors, with the inclusion of a larger pool of participants. Urrutia et al. investigated the correlation between the scores of the Modified Ashwort Scale and the measurements obtained with the Amadeo^®^ (Tyromotion, Austria) finger-hand rehabilitation device for the assessment of joint spasticity. Making a reliable and standardized robotic assessment of joint spasticity is still an open challenge due to the need to capture an intricate interaction of neurophysiological mechanisms (Pilla et al., 2020).

Longitudinal assessment protocols merging clinical evaluations with the quantitative measurement of physiological and biomechanical parameters are crucial for achieving more efficient and cost-effective rehabilitation programs. Such assessments would be extremely valuable for the stratification of patients into groups with similar characteristics to identify the most appropriate and customized treatment plan for each individual. Tesfazgi et al. analyzed the sources of uncertainty in the estimation of the human arm impedance using upper-limb wearable robotics. These uncertainties arise from the physical human–robot interaction, and their identification plays a pivotal role in the reliable and automated estimation of the user's neuromechanical state. This, in turn, can open new possibilities for true customization of rehabilitation treatments. Das et al. proposed a method for the online classification of compensatory movement strategies based on kinematic information. The automatic detection of compensatory motion could be exploited to inform the patient about the correct execution of the task, e.g., during home training without the therapist's supervision. In addition, such information could be used to adjust the assistance profiles of robotic devices to enforce the proper movement kinematics. Finally, Wu et al. demonstrated the benefits of closed-loop cueing training for people with Parkinson's disease. This strategy can provide patients with adaptive, optimized cues to improve their gait performance by learning a personalized model of the user's responsiveness to the cues.

Overall, the articles in this Research Topic provide different insights for the further development of wearable technologies across the rehabilitation continuum of care. These insights revolve around three main pillars: the need for customization of the rehabilitation treatment, the importance of an objective quantification and characterization of the patient's conditions, and the value of smart mechatronic designs to guarantee the seamless and intuitive use of wearable robotics. Further advancements in each of these three pillars will be paramount to ultimately enable the translation of wearable robotics into our daily lives.

## Author contributions

ET: Writing—original draft. SH: Writing—review and editing.
